# Genetic homogenization of indigenous sheep breeds in Northwest Africa

**DOI:** 10.1038/s41598-019-44137-y

**Published:** 2019-05-28

**Authors:** Ibrahim Belabdi, Abdessamad Ouhrouch, Mohamed Lafri, Semir Bechir Suheil Gaouar, Elena Ciani, Ahmed Redha Benali, Hakim Ould Ouelhadj, Abdelmajid Haddioui, François Pompanon, Véronique Blanquet, Dominique Taurisson-Mouret, Sahraoui Harkat, Johannes A. Lenstra, Badr Benjelloun, Anne Da Silva

**Affiliations:** 1 Science Veterinary Institute, University of Blida, BP 270, Blida, 09000 Algeria; 2 Laboratory of Biotechnology related to Animal Reproduction (LBRA), University of Blida, BP 270, Blida, 09000 Algeria; 3 National Institute of Agronomic Research (INRA Maroc), Regional Centre of Agronomic Research, Beni-Mellal, Morocco; 4 Department of Biology, Aboubakr Belkaid Tlemcen University, laboratory of Physiopathologie et biochimie de la Nutrition (PpBioNut), Tlemcen, Algeria; 5 Department of Biosciences, Biotechnologies and Biopharmaceutics, University of Bari, Bari, Italy; 6 Institut technique des Elevages Saida, Saida, Algeria; 7 Laboratoire de Biotechnologies et Valorisation des Ressources Phytogénétiques (LBVRP), Université Sultan Moulay Slimane, BéniMellal, Morocco; 8 Univ. Grenoble Alpes, Univ. Savoie Mont-Blanc, CNRS, LECA, 38000 Grenoble, France; 9 Univ. Limoges, INRA, PEREINE EA7500, USC1061 GAMAA, F-87000 Limoges, France; 10 CNRS, UMR 5815, Dynamiques du droit, Université de Montpellier, Montpellier, France; 11 Utrecht University, Faculty of Veterinary Medicine, Utrecht, Netherlands

**Keywords:** Population genetics, Animal breeding

## Abstract

Northwest-African sheep represent an ideal case-study for assessing the potential impact of genetic homogenization as a threat to the future of traditional breeds that are adapted to local conditions. We studied ten Algerian and Moroccan breeds of sheep, including three transboundary breeds, distributed over a large part of the Maghreb region, which represents a geographically and historically coherent unit. Our analysis of the dataset that involved carrying out Genome-wide SNP genotyping, revealed a high level of homogenization (ADMIXTURE, NetView, fineSTRUCTURE and IBD segments analyses), in such a way that some breeds from different origins appeared genetically undistinguished: by grouping the eight most admixed populations, we obtained a mean global F_ST_ value of 0.0024. The sPCA analysis revealed that the major part of Morocco and the Northern part of Algeria were affected by the phenomenon, including most of the breeds considered. Unsupervised cross-breeding with the popular Ouled-Djellal breed was identified as a proximate cause of this homogenization. The issue of transboundary breeds was investigated, and the Hamra breed in particular was examined *via* ROH fragments analysis. Genetic diversity was considered in the light of historical archives and anthropological works. All of these elements taken together suggest that homogenization as a factor affecting the Maghrebin sheep stock, has been particularly significant over the last few decades, although this process probably started much earlier. In particular, we have identified the policies set by the French administration during the colonial period of the region’s history as a causal factor that probably contributed significantly to this process. The genetic homogenization that we have observed calls into question the integrity of the farm animal genomic resources represented by these local breeds, whose conservation is of critical importance to the future of the livestock sector.

## Introduction

Local breeds are defined as those that have been in a country “for a sufficient time to be genetically adapted to one or more traditional production systems or environments in the country”^1^. Due to these adaptations, local breeds represent valuable genetic resources, especially in the context of climate change^2^. However, a large proportion of them are at serious risk of extinction^3^. One of the main threats is uncontrolled cross-breeding (*i*.*e*., breeding not carried out within the framework of selection plans) with more productive local or imported breeds being favoured by farmers, who face increasing economic pressure. These practices, assessed by FAO to establish risk status, lead to genetic dilution, *i*.*e*. overwhelmingly high numbers of non-local genes lowering the relative frequency of native genes in the population. They favor genetic homogenization via the loss of rare or specific variants, and induce disruption of co-adapted gene complexes, which jeopardizes the integrity and viability of the local populations^4,5^.

The stock of North African domestic sheep (*Ovis aries*) provides an ideal case-study for assessing the problem of genetic homogenization among local breeds and its impact on farm animal genetic resources. This stock is 7000 years old^6^, and contains a remarkable diversity of populations, which have been maintained under traditional farming systems over millennia^7^. These systems have enabled the emergence of breeds locally adapted. In the Maghreb, the diversity of territories (mountains, steppes, desert, oasis, *etc*.) and climates, in combination with the history of the region’s people, have shaped a rich livestock heritage.

In recent decades it has been observed that in Algeria there is a strong preference among farmers for the Ouled-Djellal breed, due to its larger conformation, and this has led to the intensification of anarchic crossing practices between local breeds. The breeders hope through such practices to increase their productivity^8–10^. This results in a strong admixture between Ouled-Djellal, Rembi, Taâdmit, and Berber breeds^11,12^. For the Berber breed, a primitive breed that is thought to be the most ancient breed of the Maghreb^13^, absorption by an Arab breed-type had already begun in 1900. Indeed, in an observation dating from that time, Couput^14^ mentions that only isolated populations, generally located in the mountains, remained preserved. Today, DNA markers do not detect any differentiation between Berber and Ouled-Djellal, Rembi or Taâdmit breeds, while the Sidaoun breed (raised by the Tuareg people), the D’Man breed (mostly encountered in oases), the Tazegzawth breed (from the Kabyle mountains) and the Hamra breed (called Beni-Guil in Morocco) all appear to be more genetically preserved^11,12^.

Our study of the genetic diversity of Maghrebin sheep relies on breeds from Morocco and Algeria, two countries that represent a substantial part of the Maghreb area. The stabilization of national borders in the Maghreb region started in the 18^th^ century. For this reason an analysis of the processes shaping these livestock, whose origins date as far back as 7000 years BP, would be better and more coherently approached under a broader geographic scale (*i*.*e*. the diversity of sheep breeds in the Maghreb should be considered at a scale above the national level). The aims of the study were to assess the genetic structuration of the Maghrebin sheep stock. Particular attention was paid to the case of the transboundary (*i*.*e*. Hamra/Beni-Guil, D’Man and Ouled-Djellal) breeds, in order to see how their history and genetic characteristics can provide insights regarding the evolution of the Maghrebin sheep stock.

## Results

A total of 180 sheep (93 from Algeria and 87 from Morocco) from ten breeds (three trans-boundary breeds, five Algerian and two Moroccan breeds, Table S1) were analyzed through a panel of 36,376 SNPs after having merged the Moroccan and Algerian data.

The average F_IS_ value was rather low (mean = −0.044, s.d. = 0.06), with 129 individuals (*i*.*e*. 71.7%) showing negative values. A total of 22 individuals showed a pronounced deficit in heterozygosity with F_IS_ values > 0.1; this deficit was particularly apparent among the Barbarine (BRBA) and Moroccan D’Man (DMNM) breeds, with 40% and 43.3% of individuals displaying such values, respectively.

Calculation of pair-wise F_ST_ (Table 1) was used to explore the genetic relationships among breeds. Two groups were detected: (i) the first group was composed of Ouled-Jellal subpopulations from Algeria and Morocco (OLDM, OLDA), D’Man from Morocco (DMNM), Hamra from Algeria (HAMA), Beni-Guil (BIGM, also called Hamra in Algeria), Berber (BERA), Sardi (SRDM), Timahdite (TMHM), and Rembi (RMBA); this group had a mean global F_ST_ value of 0.0024 [0.0021–0.0026]_95%_ and showed very low values of pair-wise F_ST_ (<0.006), with nine of these values not significantly different from zero (see confidence intervals). (ii) in the second group, which included the five Algerian remaining breeds (Sidaoun, D’Man, Hamra from pilot farms, Barbarine and Tazegzawth), the pair-wise values of F_ST_ ranged from 0.022 (Barbarine, BRBA/Rembi, RMBA) to 0.113 (Algerian preserved Hamra, HAMAP/Tazegzawth, TZGA).

**Table 1:** Pair-wise F_ST_ between Algerian and Moroccan breeds.

	SDNA	BERA	RMBA	BRBA	DMNA	OLDA	HAMA	HAMAP	TZGA	BIGM	SRDM	OLDM	DMNM
SDNA													
BERA	[0.036–0.041] 0.038												
RMBA	[0.036–0.040] 0.038	**[−0.000–0.003]** **0.001**											
BRBA	[0.059–0.062] 0.060	[0.021–0.026] 0.023	[0.021–0.024] 0.022										
DMNA	[0.041–0.044] 0.042	[0.032–0.036] 0.034	[0.032–0.037] 0.035	[0.052–0.058] 0.055									
OLDA	[0.037–0.039] 0.038	**[0.002–0.006]** **0.004**	**[−0.002–0.002]** **0.000**	[0.022–0.025] 0.023	[0.034–0.037] 0.035								
HAMA	[0.032–0.036] 0.034	**[0.001–0.004]** **0.002**	**[0.001–0.003]** **0.002**	[0.022–0.026] 0.024	[0.031–0.034] 0.032	**[0.001–0.002]** **0.001**							
HAMAP	[0.077–0.082] 0.080	[0.049–0.053] 0.051	[0.050–0.054] 0.052	[0.071–0.075] 0.073	[0.080–0.085] 0.082	[0.050–0.054] 0.052	[0.043–0.046] 0.045						
TZGA	[0.096–0.100] 0.098	[0.064–0.068] 0.066	[0.064–0.068] 0.066	[0.085–0.091] 0.087	[0.097–0.102] 0.098	[0.064–0.068] 0.066	[0.060–0.064] 0.062	[0.111–0.118] 0.113					
BIGM	[0.032–0.036] 0.034	**[−0.001–0.002]** **0.001**	**[−0.001–0.003]** **0.002**	[0.021–0.025] 0.022	[0.030–0.035] 0.032	**[0.001–0.004]** **0.002**	**[−0.002–0.001]** **−0.000**	[0.048–0.053] 0.051	[0.064–0.069] 0.067				
SRDM	[0.032–0.035] 0.034	**[0.003–0.005]** **0.004**	**[0.004–0.006]** **0.005**	[0.027–0.031] 0.029	[0.031–0.033] 0.029	**[0.005–0.007]** **0.006**	**[0.002–0.004]** **0.003**	[0.048–0.052] 0.050	[0.064–0.067] 0.065	**[0.001–0.003]** **0.002**			
OLDM	[0.034–0.037] 0.036	**[0.000–0.004]** **0.002**	**[−0.000–0.002]** **0.001**	[0.021–0.025] 0.022	[0.030–0.033] 0.031	**[0.000–0.003]** **0.002**	**[−0.000–0.002]** **0.001**	[0.048–0.052] 0.050	[0.062–0.067] 0.065	**[−0.002–0.001]** **0.000**	**[0.003–0.005]** **0.004**		
DMNM	[0.026–0.029] 0.028	**[0.001–0.003]** **0.002**	**[0.001–0.004]** **0.002**	[0.022–0.025] 0.023	[0.025–0.028] 0.026	**[0.002–0.004]** **0.003**	**[0.002–0.003]** **0.002**	[0.047–0.051] 0.049	[0.061–0.065] 0.063	**[−0.002–0.000]** **−0.001**	**[0.003–0.005]** **0.004**	**[0.000–0.003]** **0.001**	
TMHM	[0.033–0.036] 0.035	**[0.002–0.005]** **0.003**	**[0.003–0.006]** **0.004**	[0.026–0.029] 0.027	[0.031–0.033] 0.032	**[0.004–0.006]** **0.005**	**[0.002–0.003]** **0.002**	[0.045–0.049] 0.047	[0.062–0.065] 0.063	**[0.000–0.002]** **0.001**	**[0.002–0.003]** **0.002**	**[0.002–0.004]** **0.003**	**[0.002**–**0.003]** **0.002**

^*^In bold values with two zeros after the decimal point.

BERA = Berber; BRBA = Barbarine; DMNA = D’Man from Algeria; DMNM = D’Man from Morocco; HAMA = Hamra; BIGM = Beni-Guil;HAMAP = Hamra form pilot farms; OLDA = Ouled-Djellal from Algeria; OLDM = Ouled-Djellal from Morocco; RMBA = Rembi; SDNA = Sidaoun; SRDM = Sardi; TMHM = Timahdite; TZGA = Tazegzawth.

Considering the case of transboundary breeds, we found that: (i) Beni-Guil (BIGM, called Hamra in Algeria) was not differentiated from Hamra (HAMA) sampled in private Algerian farms (F_ST_ value not significantly different from zero), but was clearly differentiated from the Algerian Hamra population preserved in pilot farms (HAMAP) (F_ST_ = 0.051); (ii) D’Man populations from Morocco and Algeria (DMNM and DMNA) showed pair-wise F_ST_ of 0.026; (iii) Ouled-Djellal populations from Morocco and Algeria (OLDM and OLDA) showed pair-wise F_ST_ close to zero (F_ST_ = 0.002). The NeighborNet graph based on the F_ST_ genetic distances (Fig. S1) allows visualization of these results.

**Figure 1:**
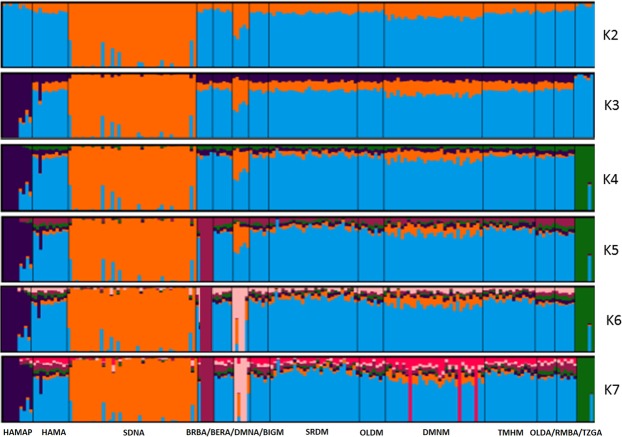
Bayesian clustering performed with ADMIXTURE software on Moroccan and Algerian sheep. K = number of clusters; HAMAP = Hamra form pilot farms; HAMA = Hamra; BIGM = Beni-Guil; SDNA = Sidaoun; BRBA = Barbarine; BERA = Berber; OLDA = Ouled-Djellal from Algeria; OLDM = Ouled-Djellal from Morocco; DMNA = D’Man from Algeria; DMNM = D’Man from Morocco; SRDM = Sardi; TMHM = Timahdite; RMBA = Rembi; TZGA = Tazegzawth.

The ADMIXTURE analysis revealed the same pattern, whereby the sheep were characterized by two groups (Fig. 1): In the “non-homogeneous” group, the Sidaoun (SDNA) breed was differentiated from all the other breeds for K = 2 (*i*.*e*. the lowest cross-validation (CV) error) and showed genetic overlap with Algerian D’Man (DMNA). Increasing K from 3 to 6, assigned clusters to the preserved Hamra population (HAMAP), Tazegzawth (TZGA), Barbarine (BRBA) and Algerian D’Man (DMNA). The second, or “homogeneous”, group was characterized by an important phenomenon of admixture, and included all of the remaining breeds. At K = 7, a few D’Man individuals from Morocco (DMNM) showed genetic distinctness. For K > 7, intra-breed structuration was also apparent for the SDNA, HAMAP, BRBA and DMNA breeds.

**Figure 2:**
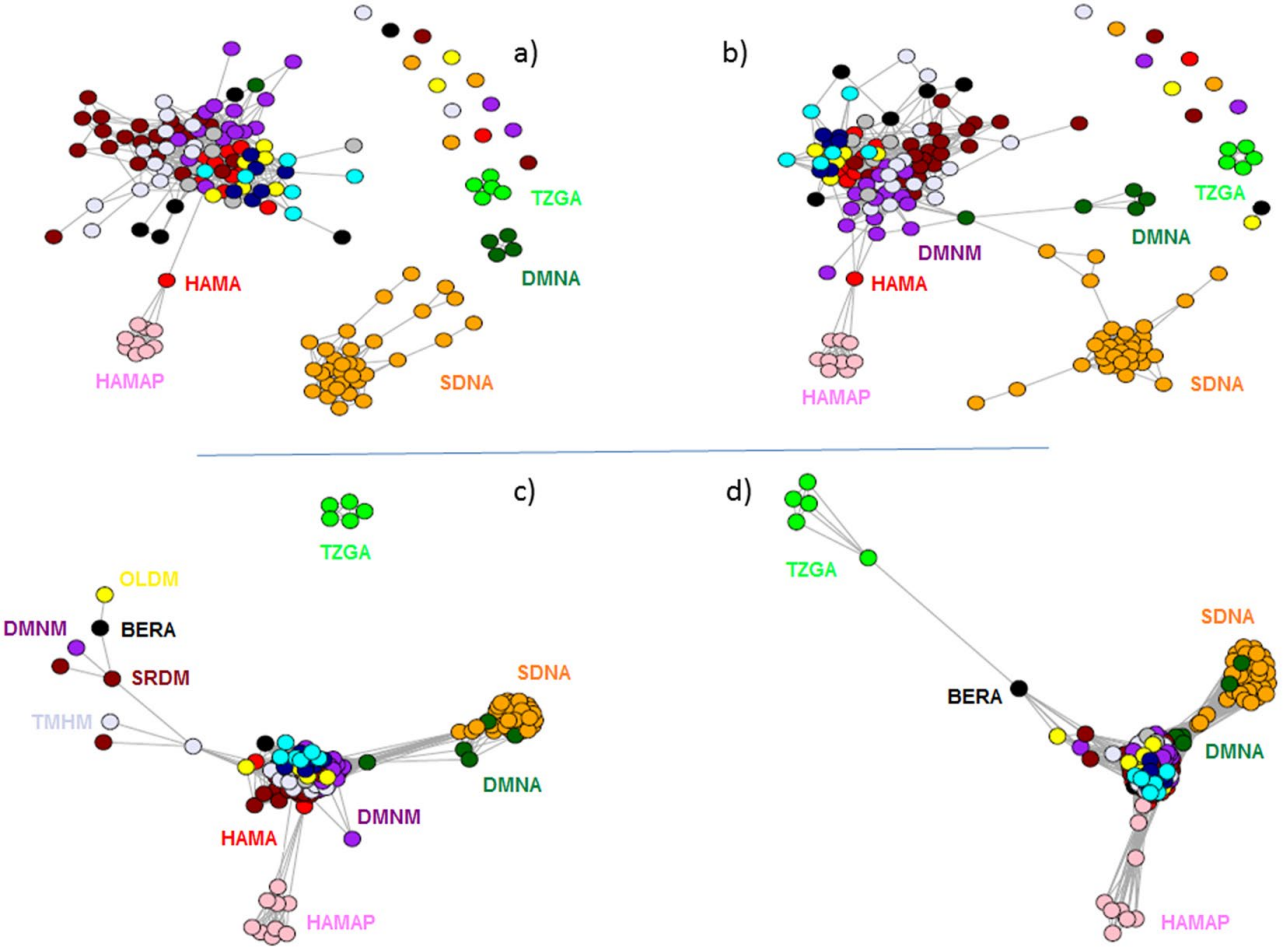
Mutual nearest-neighbour graphs obtained from NetView and considering the following k values: k = 15 (**a**), k = 25 (**b**), k = 75 (**c**) and k = 100 (**d**). Color shades represent different sheep breeds. HAMAP = Hamra form pilot farms; HAMA = Hamra; SDNA = Sidaoun; BERA = Berber; OLDM = Ouled-Djellal from Morocco; DMNA = D’Man from Algeria; DMNM = D’Man from Morocco; SRDM = Sardi; TMHM = Timahdite; TZGA = Tazegzawth.

NetView was used to represent the genetic structure of the dataset along a gradient of k values (from 2 to 100), allowing for the investigation of a range of structures, from fine scale population structures, revealed by a small value of k, to large scale population structures, revealed by a large value of k (see Fig. 2). As individuals showing F_IS_ value > 0.1 were removed from the analysis (see)^15^, we were led to remove the Barbarine (BRBA) breed, which was then only represented by three individuals. The simulated data network was based on a minimum spanning tree. At fine scale (Fig. 2a, k = 15) the topology of the network showed the existence of four homogeneous clusters corresponding to Sidaoun (SDNA), Algerian D’Man (DMNA), Tazegzawth (TZGA) and Algerian preserved Hamra (HAMAP). It was interesting to note that HAMAP was closely linked to one HAMA individual (Algerian Hamra sampled in private farm). Most of the other individuals were highly interconnected. At k = 25 (Fig. 2b), Algerian D’Man (DMNA) was connected (i) with the central and admixed core, *via* a Moroccan D’Man (DMNM) individual, (ii) and on another side, with the Sidaoun (SDNA) cluster. At k = 75 (Fig. 2c), clear connections appeared between Algerian D’Man (DMNA) and Sidaoun (SDNA); moreover only Tazegzawth (TZGA) individuals remained unlinked to the central core. The connection of TZGA to the central core was only observed at k = 100 (Fig. 2d) and *via* a Berber (BERA) individual (*i*.*e*. a breed reared in Kabyle mountains as TZGA). The tree based on Allele-Sharing Distance (ASD) distance between individuals (Fig. S2) also showed genetic relatedness between a number of individuals belonging to Sardi and D’Man breeds (*i*.*e*. Moroccan breeds).

**Figure 3:**
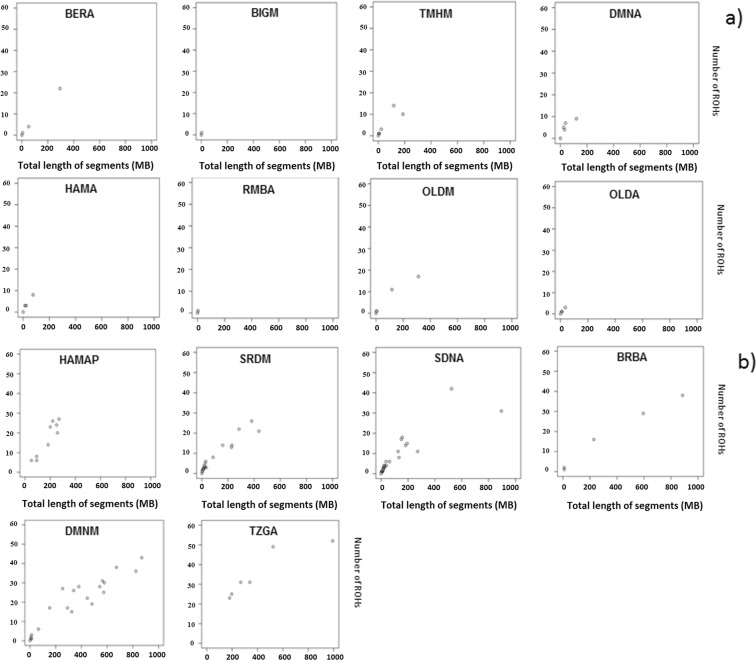
Runs of homozygosity (ROH) identified for Moroccan and Algerian sheep breeds. The number of ROHs found for each individual genome (y-axis) is plotted against ROH total size (i.e. the number of Kb covered by ROH in each genome, x-axis); (**a**) breeds in which ROH length was close to zero, (**b**) others. HAMAP = Hamra form pilot farms; HAMA = Hamra; BIGM = Beni-Guil; SDNA = Sidaoun; BRBA = Barbarine; BERA = Berber; OLDA = Ouled-Djellal from Algeria; OLDM = Ouled-Djellal from Morocco; DMNA = D’Man from Algeria; DMNM = D’Man from Morocco; SRDM = Sardi; TMHM = Timahdite; RMBA = Rembi; TZGA = Tazegzawth.

**Figure 4:**
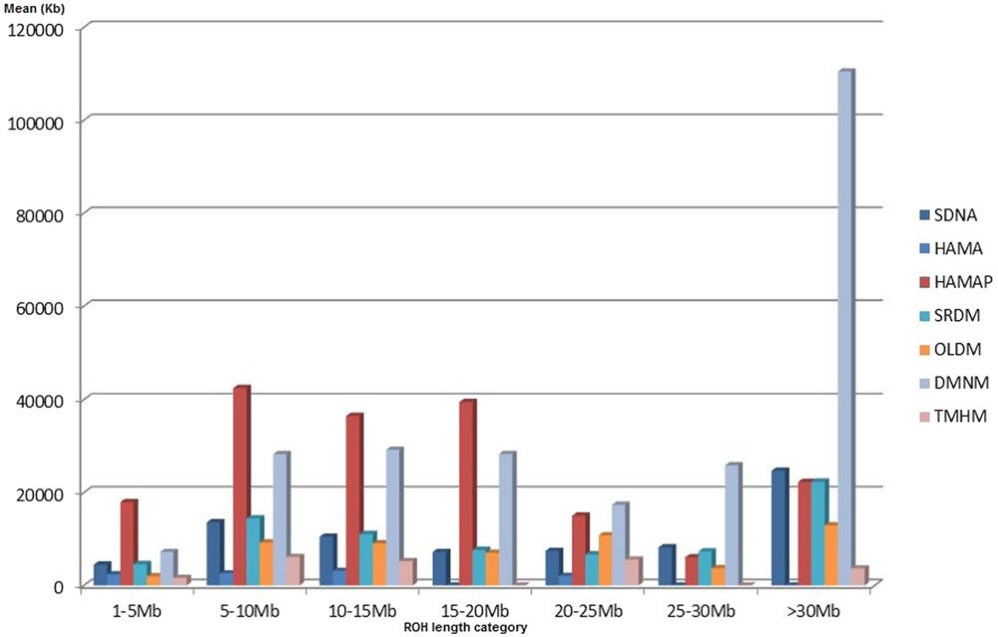
Classification of ROHs in seven categories (x-axis) according to size (from 1 to 5 Mb to more than 30 Mb) and mean sum of ROHs (y-axis,measured in kilobases) within each ROH category and averaged per breed. We analyzed sheep breed from Algeria and Morocco. Results were not presented for breed with sample size ≤6. HAMAP = Hamra form pilot farms; HAMA = Hamra; SDNA = Sidaoun; OLDM = Ouled-Djellal from Morocco; DMNM = D’Man from Morocco; SRDM = Sardi; TMHM = Timahdite.

The characteristics of ROH, were used as source of information about demographic history. Given the limited size of some samples, the purpose of the ROH and IBD segment analyses (see next paragraph) was not to study each breed according to the values obtained, but to assess the extent to which strong patterns, *i*.*e*. clear splits between groups of breeds, were also highlighted by these methods. Indeed, plotting the number of ROHs found for each individual genome against ROH total size (Fig. 3) revealed a clear separation between two groups: in the first group (Fig. 3a) of eight breeds (*i*.*e*. a group containing the DMNA breed plus all breeds, except DMNM and SRDM, that were identified by the previous analyses of F_ST_, Admixture and NetView as belonging to the “homogeneous” group), most individuals carried few and short ROHs. The 50 K SNP panel did not detect any ROH in Beni-Guil (BIGM, called Hamra in Algeria), Hamra sampled in private Algerian farms (HAMA), Algerian Ouled-Djellal population (OLDA) and Rembi (RMBA). Such profiles are characteristic of genetically diverse and most probably admixed populations^16,17^. The second group (Fig. 3b), included Barbarine (BRBA), Tazegzawth (TZGA), the preserved Algerian Hamra population (HAMAP), Sidaoun (SDNA) (*i*.*e*. all of the breeds identified by the previous analyses, F_ST_, Admixture and NetView, as belonging to the “non-homogeneous” group, except DMNA), and also Sardi (SRDM), and the Moroccan D’Man population (DMNM). This second group was characterized by a higher number of ROHs. Tazegzawth (TZGA) showed several long ROH fragments, a pattern characteristic of recent inbreeding, resulting in F_ROH_ > 0.1 for four individuals out of the six considered (Table S2). Similarly, long ROH fragments were detected in Barbarine and F_ROH_ showed very high values (*i*.*e*. 0.22 and 0.34) for two of the five individuals. The Hamra subpopulation, preserved in Algerian pilot farms (HAMAP), received special attention through the analysis of the ROH distribution according to their size (Fig. 4). For this breed, ROH showed a rather uniform distribution for the short and intermediate length categories. Such a pattern is characteristic of an isolated population that is based on a source population of quite large size, whereas a different pattern showing an overrepresentation of short ROHs would have been the signature of a strong population bottleneck^18,19^. Moreover, the low abundance of long ROHs suggests that management practices have been used to avoid inbreeding for HAMAP, which was confirmed by the F_ROH_ values (Table S2). We further noted recent inbreeding for the Moroccan D’Man population (DMNM), indeed ROH size distribution was relatively uniform for classes between 5 and 25 Mb whereas a peak revealed very high values for extremely long ROHs (>30 Mb). The F_ROH_ consideration indicated that 43% of individuals showed a high value (*i*.*e*. F_ROH_ > 0.1) (Table S2).

IBD segments analysis (Table 2) revealed that all the breeds except Tazegzawth (TAZ), the preserved Hamra population (HAMAP) and the Algerian D’Man population (DMNA), were characterized by absence or quasi absence of IBD segments. It is worth noting that Sidaoun (SDNA) also contained almost no IBD segments. For comparison purposes, similar analyses were conducted on Italian local breeds (*i*.*e*. breeds standardized during the 20^th^ centuries) and South-West Asian breeds (see Supplementary Text 1 and 2) highlighting a clear relationship between the occurrence of admixture and a decrease, in number and length, of IBD segments.

**Table 2:** Number and lengths of IBD segments.

Breeds	Nb. of animals	Nb. of IBD segments	Nb. of IBD segment/Nb. animals	% IBD segment intra-breed	% IBD segment inter-breed	Mean length (SD) Mb
Hamra from Algerian pilot farms	10	456	45.6	100	0	14.11 (11.14)
Tazegzawth	5	245	49*	100	0	14.92 (9.87)*
D’Man from Algeria	5	19	3.8*	100	0	14.06 (8.50)*
Ouled-Djellal from Algeria	6	1	0.17*	100	0	4.99 (−)*
Sidaoun	36	4	0.11	100	0	9.91 (5.19)
Sardi	24	1	0.04	100	0	8.45 (−)
Timahdite	16	7	0.44	29	71	7.15 (1.78)
Berber	6	0	—	—	—	—
Beni-Guil	6	0	—	—	—	—
Barbarine	3	0	—	—	—	—
D’Man from Morocco	17	0	—	—	—	—
Hamra	10	0	—	—	—	—
Ouled-Djellal from Morocco	8	0	—	—	—	—
Rembi	6	0	—	—	—	—

Nb., number of individuals considered (*i*.*e*. individuals showing F_IS_ values > 0.1); SD, Standard Deviation; *values given as an indication, since the limited size of the samples biases the average estimates.

**Figure 5:**
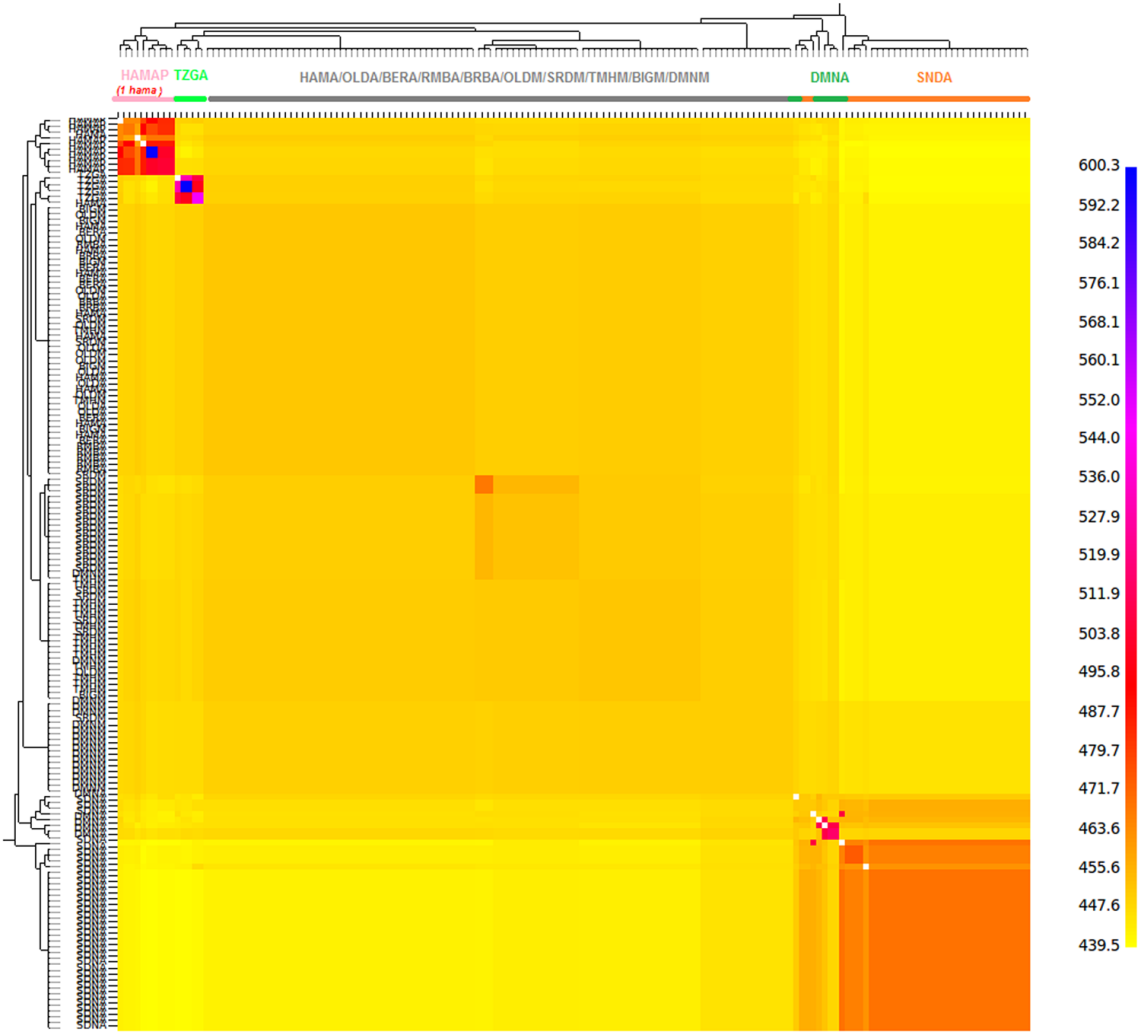
FineSTRUCTURE clustering for Algerian and Moroccan sheep breeds. The color of each bin in the matrix indicates the number of “genomic chunks” copied from a donor (column) to a recipient individual (row). HAMAP = Hamra form pilot farms; HAMA = Hamra; BIGM = Beni-Guil; SDNA = Sidaoun; BRBA = Barbarine; BERA = Berber; OLDA = Ouled-Djellal from Algeria; OLDM = Ouled-Djellal from Morocco; DMNA = D’Man from Algeria; DMNM = D’Man from Morocco; SRDM = Sardi; TMHM = Timahdite; RMBA = Rembi; TZGA = Tazegzawth.

The co-ancestry heatmap (Fig. 5) obtained with CHROMOPAINTER/fineSTRUCTURE presents the number of shared haplotypes (“chunks”) between Algerian and Moroccan individuals. As recommended by Van Dorp *et al*.^20^ individuals showing high values of F_IS_ (>0.1) were excluded from the analysis. The individuals belonging to the Sidaoun (SDNA) and Algerian D’Man (DMNA) breeds clustered and were separated from the other populations on the basis of shared haplotypes. The Tazegzawth (TZGA) and Algerian preserved Hamra (HAMAP) breeds (and one HAMA sheep, *i*.*e*. the same individual who was identified in the Netview analysis, Fig. 2) were also clearly individualized as animals showing the highest number of genomic shared chunks in the dataset, whereas the remaining breeds appeared clearly admixed. When considering Sidaoun (SDNA) in more detail, it turns out that the number of chunks shared within the breed was significant but that these shared haplotypes were also characterized by their relatively small size (matrix of chunk length not shown), which accounts for the fact that the previous IBD analysis did not detect them (Table 2).

**Figure 6:**
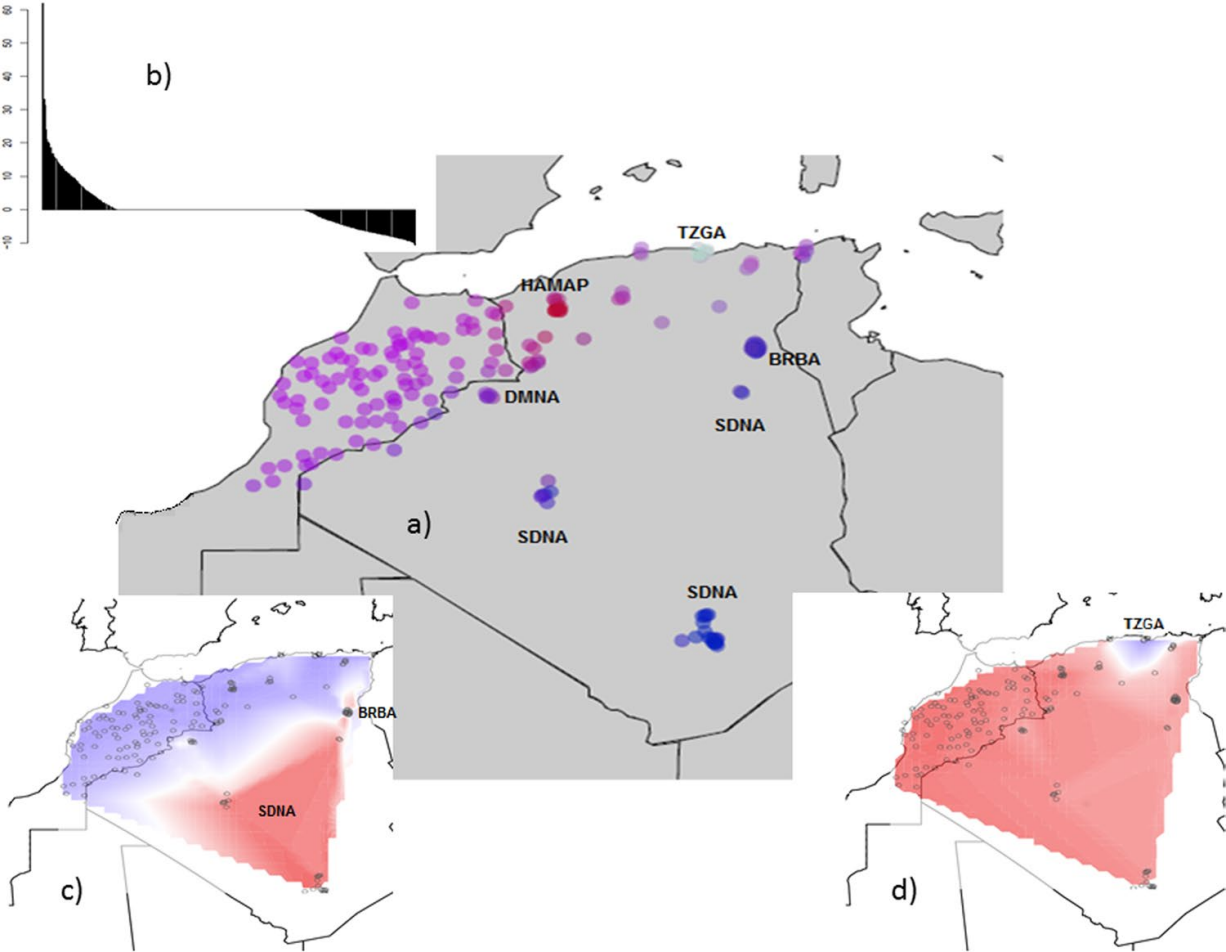
Spatial PCA (sPCA) Analysis of Algerian and Moroccan sheep breeds (**a**); eigenvalues for each global and local axis (positive and negative eigenvalues indicate global and local structures, respectively) (**b**); first global score of sPCA (**c**); second global score of sPCA (**d**). HAMAP = Hamra form pilot farms; SDNA = Sidaoun; BRBA = Barbarine; DMNA = D’Man from Algeria.

In the spatial PCA analyses using Delaunay triangulation as connection networks, the consideration of eigenvalues suggested the possible occurrence of a spatial pattern, as the three first positive scores were distinguished from other eigenvalues (Fig. 6b). The global Monte Carlo test (10,000 iterations) indicated significant global spatial structure (p-value = 0.009). The Mantel test showed no significant correspondence between geographic and genetic distances (p-value = 0.07). Indeed, while the north constituted a largely homogeneous block, individuals separated by more than 2000 km appeared very close from a genetic point of view. The eigenvectors of the three first global scores plotted according to the geographical coordinates indicated a clear homogeneity of the Northern stock (*i*.*e*., Moroccan and Northern-Algeria sheep) with the Hamra preserved in pilot farms (HAMAP) and Tazegzawth (TZGA) as exceptions (Fig. 6a). The first coordinate highlighted the contrast of Sidaoun (SDNA) and Barbarine (BRBA) with the other breeds (Fig. 6c) and the second coordinate highlighted the separate position of the Tazegzawth (TZGA) (Fig. 6d).

## Discussion

This study provides the opportunity to assess genetic diversity in several traditional sheep breeds in the Maghreb, a region which constitutes a coherent unit from geographical and historical perspective.

All of the analyses revealed a high level of genetic homogenization for most breeds, irrespective of their origin. More specifically, the breeds affected by this phenomenon were Ouled-Djellal (populations from Morocco and Algeria), Timahdite, Sardi, Hamra/Beni-Guil (populations from Morocco and Algerian private farms, but not those from Algerian state farms), D’Man (the population from Morocco but not the population from Algeria), Rembi and Berber. We observed a strong spatial pattern with two major areas identified. The first one was the Northern area, corresponding to the major part of Morocco and the Northern part of Algeria delimited to the South by steppe. This area appeared homogeneous for all the breeds previously mentioned except for two differentiated breeds, *i*.*e*. the Hamra breed preserved in pilot farms and the Tazegzawth breed from the Kabyle Mountains. The second area identified was the Southern area, including breeds from the Sahara and Oriental Great Erg regions, *i*.*e*. the Sidaoun and Barbarine breeds. This second area appeared clearly differentiated from the Northern one.

Chromopainter analysis showed that breeds in the homogeneous group, clustered together and shared few genetic chunks (haplotypes). The characteristics of ROHs, used as sources of information about the demographic history of the breeds^18,19,21^ gave rise to consistent conclusions. Indeed, most of the breeds mentioned above were characterized by a very limited number of ROHs with very small sizes. In particular, four of them, Rembi, Ouled-Djellal from Algeria and Beni-Guil/Hamra from Morocco and private Algerian farms, were characterized by the quasi absence of ROH, clearly indicating admixture^16,17^. Moreover, these breeds were characterized by extremely few or no IBD segments, what was shown to also be a signature of genetic homogenization (see Supplementary Text 1 and 2). Indeed, it seems likely that strong and continuous admixture, in small ruminants (characterized by relatively short generation time) breaks up contiguous genomic blocks resulting in very small haplotypes.

Among the breeds identified as being genetically differentiated from other breeds, our analysis appeared to show that the Tazegzawth breed, reared by Kabyle people in geographically isolated conditions, is threatened by inbreeding (see ROH analysis). These results were not surprising given that the breed accounts for less than 0.02% (*i*.*e*. a few hundred heads) of the present-day Algerian sheep stock. In contrast, the Sidaoun breed, reared by Touareg people, did not appear to be affected by inbreeding. This breed was genetically differentiated from the other studied breeds (as shown by *e*.*g*. sPCA, Admixture, NetView or F_ST_ analyses), but would have experienced a significant level of admixture with breeds/populations not considered in this study. Indeed, the Chromopainter and IBD segments analyses reveal a very particular profile for this breed with quite a large number of intra-breed shared segments, but with such segments characterized as being relatively small in size. Further investigations will be needed, for example focusing on the genetic relationship between this breed and the Malian sheep stock, which is thought to be the cradle of the Sidaoun breed. Another southern breed, the Barbarine, a transboundary breed related to the Tunisian Barbarine (*i*.*e*. the main breed in Tunisia), showed clear genetic distinctness. This fat-tail breed originates from West-Asia, which may explain its genetic distance from all other breeds considered in this study. Analyses of ROH (distribution and F_ROH_) raised concerns about possible inbreeding problems, however, only examination of a larger sample could lead to robust conclusions. Such conclusions would not be surprising if we consider the size reductions suffered by the Algerian Barbarine population in recent decades. Indeed, whilst the fat tail of the Algerian Barbarine sheep means that the animal is remarkably well adapted to arid environments, this same trait hinders the animal’s marketability^22^, its slaughter quality being considered as diminished because of it (the amount of caudal fat can be 2 to 4 kg, which is not marketable). The Algerian Barbarine breed has therefore lost the interest of breeders, who have abandoned it in favor of the Ouled-Djellal breed^23^. The effect of this preference among Algerian breeders is so significant that the analysis of information contained in the DAGRIS database (http://dagris.ilri.cgiar.org/) now indicates that the Barbarine breed is in danger of being “absorbed by Ouled-Djellal” in Algeria.

Focusing on transboundary breeds, it is interesting to note that the genetic specificity of Algerian D’Man, sampled from state farms that have been tasked with breed preservation, was not observed in Moroccan samples, which were collected in private farms. In the Moroccan population, many very long stretches of ROH revealed strong levels of recent inbreeding for a number of individuals. This breed, which is highly adapted to oasis environments and is encountered in Morocco and Algeria, has a probable Moroccan origin. Indeed, its Algerian name is “mouton Filali” suggesting that the cradle of the breed could well be Tafilalet (in Morocco). The French agronomist G. Toutain^24^, lamented, even as far back as 1979, the lack of due attention given to this breed, which he qualified as a “genetic treasure” shaped by traditional practices over centuries. In particular, when making those comments Toutain regretted that the Merino breed was being considered as good candidate to introduce in the oases. An investigation of archive records might provide a way to trace the history of this breed and thereby understand the process that led to the breed’s present-day genetic dilution.

In our study another transboundary breed, Hamra/Beni-Guil, was sampled from different sources, with samples taken both in Morocco and in Algeria, and in different types of farms (pilot farms that have been tasked with preserving the breed, and private farms). The study of this breed through these different populations has been particularly instructive. Among the three sample sites, only sheep reared in the Algerian pilot farms appeared not to be genetically diluted (with also one individual sampled in an Algerian private farm). The main state farm in charge of the preservation of the Hamra breed (Algeria), gathered its initial herd at the end of the 1980s. Since then, a few carefully selected progenitors from private farms have occasionally been introduced into the initial core, over the years. Moreover, a strict breeding program was devised to prevent inbreeding. All the genetic results were in accordance with the management practices reported for the pilot farm as they showed (i) a pattern of short to intermediate ROH sizes, which is a pattern specific to that of a population that is isolated but that is nonetheless based on a source population of significant size^18,19^ (*i*.*e*. not a pattern that indicates a strong bottleneck effect), (ii) a low abundance of long ROHs suggesting management practices avoiding inbreeding. Taking all of these elements into account, we can assume that Hamra’s dilution has probably intensified over the past 30 years. Indeed the pilot state farms were established at that time, yet they have visibly allowed for the preservation of at least part of the genetic specificity of the breed, even if the founding effect experienced when the farm was created undoubtedly led to a reduction in genetic diversity. This hypothesis is largely supported by various anthropological studies. These have shown that over the last few decades there has been a significant intensification of crossing practices between Ouled-Djellal and this breed in private farms: Bechchari *et al*.^25^ described an “invasion phenomenon” characterized by a growing number of Ouled-Djellal in Beni-Guil herds. According to interviews conducted by Bechchari *et al*.^25^, it seems that the expansion of the Ouled-Djellal breed is mainly the result of the sedentarization and intensification of agriculture, *i*.*e*., breeders, who are under increasing pressure to improve productivity, favor Ouled-Djellal for its market value. These results were confirmed by Brisebarre^26^ who, moreover, reported massive inflows of Ouled-Djellal originating from Algeria. A figure of 200,000 to 500,000 heads of Ouled-Djellal (depending on the interviewed source) was estimated have been illegally entering Morocco each year^26^.

If we consult the historical archives, we learn that it has long been recognised that the Hamra breed is an ancient one, belonging to a stock that was passed down within a tribe from one generation to the next, and that this practice likely originated with the Zénète people. The breed’s geographic isolation is also related to the specific geological features of the Oran plateau where it lives^27^. The strong local adaptation of this breed, with respect to climate, vegetation and disease resistance, was emphasised as far back as 1891, in a report commissioned by the General Governor of Algeria^28^, which concluded that introduction of other breeds to this territory and the crosses induced would jeopardize the survival capacity of the Hamra breed.

Moreover, it may be noted that this breed, called “Le petit Oranais” by the settlers, was particularly appreciated in France for its organoleptic qualities and conformation. This breed of sheep was therefore particularly affected by the sheep export activities in Algeria, with the animals transported live by boat. At the end of the 19th century, it is estimated that this trade, from Algeria to France^29^, involved more than one million sheep heads per year (all breeds considered). Today, the main pilot farm responsible for the breed’s preservation keeps 600 sheep or 1% of the total number of Hamra heads in Algeria. Since 1980 the Moroccan government has implemented countrywide a so-called “sheep strategy”, under which most of the country has been divided into cradles of the main breeds. In these areas, breed phenotypic standardization was highly favored and measures were taken to limit gene flows^30^. Our study did not identify any preserved areas from a genetic point of view. However, it should be stated that our sampling design in Morocco did not focus on the cradle of breeds, contrary to the Algerian sampling.

If we refer to the historical archives, it appears that the colonial period in the region’s history probably contributed significantly to the expansion of the Ouled-Djellal breed. Indeed, at the end of the 19^th^ century, studies commissioned by the colonial government concluded that the “white breed” of the steppes was the breed on which all efforts should focus^31,32^. The aim of the settlers was to drastically increase the number of sheep by promoting this breed through various means (steppe development and irrigation works, creation of “model” farms, *etc*.), with guidelines explicitly recommending the elimination of breeds considered useless, of poor quality, or judged “abnormal” with regard to their conformation (the Berber and the Barbarine breeds were the ones that were mainly affected by such guidelines).

The diversity pattern of the Maghrebin sheep appears very similar to that revealed by Ouchene-Khelifi *et al*.^33^ in their study looking at the Maghrebin goat in the same area. Indeed, the results of this goat study also showed very weak genetic structuration among Moroccan breeds (*i*.*e*. Black, Draa, Nord) and the most widespread of the Algerian breeds (*i*.*e*. Arabia). Despite the breeds being from different geographical origins (*i*.*e*. Black from Morocco is Berber type and Arabia from Algeria is Sahelian type), they appeared quite indistinct from each other from a genetic perspective (*i*.*e*. mean F_ST_ was of 0.005 [0.004–0.006]_IC95%_ considering the four breeds together). Two breeds were distinguished from this main homogeneous group: the Algerian ancestral Kabyle and M’Zabite breeds, which have both been reared in isolated conditions, due to social and political factors, since ancient times. In order to assess how the patterns observed in Maghrebin small ruminants were influenced by migration processes, outward from centers of domestication, we examined the results provided by Colli *et al*.^34^ for goats. Considering the 12 African countries for which at least two local breeds were genotyped by country (*i*.*e*. a total of 37 breeds, distributed from north to south across the continent), it can be seen that of the 703 pair-wise F_ST_ calculated, only 12 showed values lower than 0.01 and none of them had a value lower than 0.006. These results postulate for a clear structuring of African goat breeds, whatever the migration routes considered, with occasional cases of more intense gene flows, generally occurring between two breeds. The data currently available do not allow for a study looking specifically at breeds of sheep in Africa. However, Gaouar *et al*.^12^ observed that, out of a total of 4460 pair-wise F_ST_ values, and considering Algerian and worldwide breeds (from Kijas *et al*.^35^) all together, instances of particularly low pair-wise F_ST_ values were only found for Algerian breeds and Australian Merino populations (*i*.*e*. strains of Merino that are not breeds as such). Thus, to our knowledge, as things currently stand, it seems unlikely that the genetic homogenization identified is an effect that results from ancestral migration processes (given that the F_ST_ values are outside the norm and affect no less than seven breeds, in a geographical area covering more than several hundred thousand km²).

Maghrebin breeds are representative of traditional breeds more generally, that, worldwide, are a cornerstone of the rural economies on which smallholder farmers and herders rely. These Maghrebin breeds were not standardized during the 20^th^ century, unlike European breeds, and therefore they continue to show intra-breed phenotypic diversity. They could be qualified as landrace breeds, representing an initial stage of breed development, with a genetic specificity mainly resulting from the combination of founder effects, isolation and natural selection. As emphasized by Sponenberg *et al*.^36^ isolation, resulting from geographic factors, but also from social practices (*e*.*g*. tribe organization) and political contexts, represents a key factor for the development and the preservation of the genetic integrity of landrace breeds. In the Maghreb, such isolation has been disturbed, inducing the genetic homogenization observed in most Maghrebin sheep breeds. In recent decades, motorized transport has certainly played an important role in increasing long-distance gene flow.

In conclusion, our study highlights a strong homogenization of the Maghreb sheep stock affecting the major part of Morocco and most of Northern Algeria. We still lack information regarding the scale at which this phenomenon of homogenization might be occurring worldwide. However, the observation of such a phenomenon clearly calls into question the integrity of farm animal genomic resources represented by these local breeds, whose conservation is of critical importance for the development of livestock breeding in the context of global changes^2^.

## Material and Methods

### Ethics statement

Samples were collected by veterinarians during routine blood sampling, for medical care or follow up, such that no ethical authorization was required. All the samples and data processed in our study were obtained with the breeders and breeding organizations’ consent.

### Breeds

We studied ten Algerian and Moroccan breeds representing most of the local breeds for this area. Three transboundary breeds were sampled in both countries: Ouled-Djellal, Hamra (Algerian name)/Beni-Guil (Moroccan name) and D’Man. Most of the breeds were “wooled thin-tailed sheep”, but the D’Man breed belongs to the “mixed hair-wool sheep” group, Sidaoun to the “hairy sheep” group and Barbarine was the only “fat-tailed” breed in our study. Algerian breeds were described by Gaouar *et al*.^11,12^. Supplementary Table 1 provides essential information about the breeds studied.

The geographical distribution of the breeds based on their cradle of origin, as described in the 1990s by Chellig^37^ for Algeria and by Boujenane^38^ for Morocco, is presented in Fig. S3. This distribution reflects a pattern described by French settlers at the beginning of the 19^th^ century, responsible for drawing up an inventory of the country’s wealth. In addition, the consultation of several historical archives^14,24,27,28,31,32,39–41^ made it possible to record essential information about these landrace breeds, which have been extensively described by zootechnicians and agronomists, including: (i) phenotypic traits specific to the different breeds (neckline shape, coat colour, wool quality, *etc*.); (ii) detailed descriptions of the areas occupied by the breeds (vegetation, topography, *etc*.), and of the geographical factors implying a more or less extensive isolation of the populations; (iii) history and breeding practices of the tribes that have maintained the traditional breeds; (iv) crossbreeding experiments undertaken by the settlers with imported breeds but also between local breeds. A general observation was the strong consistency of the genetic material within the indigenous breeds as evidenced by the heritability of the characteristic traits.

### Sampling design

The whole dataset included 46 Algerian samples from Gaouar *et al*.^12^, 87 Moroccan samples provided by the NextGen project (Grant Agreement no. 244356), plus 47 Algerian samples genotyped specially for this study (*i*.*e*. 14 additional samples of the Hamra breed and 33 additional samples of the Sidaoun breed). If we consider the case of the Hamra breed: previously available genotypes^12^ (n = 6) were only representative of one Algerian pilot farm (Aïn El Hadjar). With the further sampling, 3 samples from two other Algerian state farms and 11 samples from private farms were added to the dataset. The sampling design was developed in such a way that it was possible to study the trajectories of different populations of the same breed. The sheep were sampled, as much as possible, from different flocks in order to limit relatedness among individuals and optimize representativeness of breeds. In cases where several samples were obtained from the same farm, the choice was based on the pedigree documentation (if available) and/or genealogical information provided by the breeder. For a few breeds only few samples were available, but this did not affect the robustness of the results, as almost all analytical methods used were based on the consideration of individuals (the very few calculations based on average values were reported only as being of indicative value in the case of small samples). Moreover, we used the Weir & Cockerham *F*_ST_ estimator for quantifying differentiation between breeds, since in contrast to the Reynolds’ distance it is not significantly biased by a small sample size provided that, as in our case, the number of markers used is large^42,43^.

### Genotyping and SNP quality control

Blood samples were cryo-preserved until DNA extraction and analysis were carried out. Genomic DNA was purified from whole blood by protease K digestion and a salting-out procedure^44^. All Algerian animals were genotyped for 54,241 SNPs, using the Illumina® Ovine SNP50 BeadChip (Illumina, Inc.), following standard operating procedures recommended by the manufacturer. Genotyping was performed by the Van Haeringen Laboratorium (Wageningen, The Netherlands). Moroccan SNP data were extracted from the WGS variation using the Ovine SNP50 BeadChip coordinates of SNPs on the OAR v3.1 reference genome assembly using Vcftools^45^. High concordance between 12× re-sequencing data and the Ovine SNP50 BeadChip was demonstrated in sheep by Benjelloun *et al*.^46^. Moroccan and Algerian datasets were merged, and SNPs and animals were pruned with PLINK v1.07^47^ using the following filtering out parameters: (i) SNP call rate ≤ 97%, (ii) SNP minor allele frequency (MAF) ≤ 1%, (iii) animals displaying ≥ 10% of missing genotypes.

### Data analysis

#### Genetic diversity, runs of homozygosity and segments of identity-by-descent (IBD)

Inbreeding coefficient (F_IS_) per individual was assessed in each breed using PLINK. Runs of homozygosity (ROHs) were identified with PLINK using a sliding windows approach. The fraction of 50 SNP-windows that were almost completely homozygous (*i*.*e*. allowing: one heterozygous SNP, five missing genotypes and a maximum gap of 1000 kb) was calculated following recommendations of Manunza *et al*.^19^. Indeed, it has been shown that this approach (*i*.*e*. considering windows of SNPs that are not completely homozygous) is more powerful in detecting truly autozygous segments^48^. To reduce the occurrence of spurious runs of homozygosity (ROHs), the minimum number of SNPs required to constitute a ROH was estimated using the method proposed by Lencz *et al*.^49^. The inbreeding coefficient based on ROH (F_ROH_)^50^ was calculated by dividing total ROH length per individual by total autosomal SNP coverage (2.44 Gb).

BEAGLE 4.1^51^ was used for detection of IBD segments. The ibdtrim parameter was set to 40. Segments with a LOD score < 4 and a length shorter than 0.5 cM were excluded. Individuals showing F_IS_ values > 0.1 were excluded from analyses.

#### Genetic structure

The extent of population subdivision was examined by calculating pair-wise F_ST_ values of Weir and Cockerham^52^ and the associated 95% confidence intervals using Genetic Data Analysis (GDA) software^53^.

We used the network-based approach implemented in NetView v.1.1^15,54^ available in R. This visualization tool allows for the analysis of complex genetic structure *via* the construction of population networks through mutual k-nearest neighbours thresholds applied to genome-wide SNPs. PLINK was used to construct the initial ASD matrix (–distance-matrix, *i*.*e*. 1 - Identity by Similarity). As recommended by Neuditschko *et al*.^15^, the dataset was first subjected to quality control (*i*.*e*. minor allele frequency (<0.01) and significant deviation from Hardy–Weinberg equilibrium (P < 0.001)). We explored the data in a range of k (*i*.*e*. parameter determining the number of mutual nearest neighbours) from 2 to 100 that was determined following recommendation of Neuditschko *et al*.^15^ with the “selection plot” function.

A tree based on ASD distance between individuals and a NeighborNet graph based on F_ST_ genetic distance were constructed using Splitstree^55^.

ADMIXTURE software^56^ was used to investigate the relationship between sheep breeds. ADMIXTURE was run for K = 2 through K = 14, and ten independent runs were performed for each value of K. The program CLUMPAK^57^ available at http://clumpak.tau.ac.il, was used to analyze the multiple independent runs at a single K and visualize the results.

The approach of fineSTRUCTURE^58^ based on haplotypes was also used. The dataset was filtered and phased using SHAPEIT ver. 2^59^. We used CHROMOPAINTER^58^ to analyze the painted dataset in order to identify homogenous clusters. Visualization of the posterior distribution of clusters was then performed using the tree-building algorithm of fineSTRUCTURE.

Spatial analysis of Principal Components (sPCA) was performed with the R^60^ package ADEGENET^61^, using the Delaunay triangulation as connection network^62^. Monte Carlo tests were used to check the statistical significance of spatial structures (global and/or local spatial structure) for 10,000 iterations. The sPCA results were visualized by plotting the samples according to their geographic coordinates and coloring them according to their respective scores along the third first sPCA components.

## Supplementary information

Supplementary Information

## Data Availability

Genotypes obtained with the Illumina Ovine SNP50 BeadChip (genotype files and markers files by chromosomes) for Sidaoun and Hamra breeds in a Plink format are available from the Dryad Digital Repository, 10.5061/dryad.24p1k82.
