# Supplementary material for: Genetic homogenization of indigenous sheep breeds in Northwest Africa
Source: Sci Rep. 2019 May 28;9:7920. doi: 10.1038/s41598-019-44137-y (PMC6538629; doi:10.1038/s41598-019-44137-y)
Supplement: Supplementary file 1 — Supplementary Information [file 41598_2019_44137_MOESM1_ESM.pdf]

Supplementary Information

Genetic homogenization of indigenous sheep breeds in Northwest Africa

Ibrahim Belabdi[1,2ɣ]; Abdessamad Ouhrouch[3,7ɣ]; Mohamed Lafri[1,2]; Semir Bechir Suheil Gaouar[4]; Elena Ciani[5]; Ahmed Redha Benali[1,2]; Hakim Ould Ouelhadj[6]; Abdelmajid Haddioui[7]; François Pompanon[8]; Véronique Blanquet[9]; Dominique Taurisson-Mouret[10]; Sahraoui Harkat [1,2]; Johannes A. Lenstra[11]; Badr Benjelloun[3,8#] ; Anne Da Silva[9#*]

*Correspondence: Anne Da Silva, Fax: +33 (0)555 45 76 53, E-mail : anne.blondeau@unilim.fr

[ɣ]: Contributed equally to this work

[#]: Contributed equally to this work

[1]: Science Veterinary Institute, University of Blida, BP 270, Blida 09000, Algeria

[2]: Laboratory of Biotechnology related to Animal Reproduction (LBRA), University of Blida, BP 270, Blida 09000, Algeria

[3]: National Institute of Agronomic Research (INRA Maroc), Regional Centre of Agronomic Research, Beni-Mellal, Morocco

[4]: Department of Biology, Aboubakr Belkaid Tlemcen University, Tlemcen, Algeria, laboratory of Physiopathologie et biochimie de la Nutrition (PpBioNut)

[5]: Department of Biosciences, Biotechnologies and Biopharmaceutics, University of Bari, Bari, Italy.

[6]: Institut technique des Elevages Saida, Algérie.

[7]: Laboratoire de Biotechnologies et Valorisation des Ressources Phytogénétiques (LBVRP), Université Sultan Moulay Slimane, BéniMellal, Maroc.

[8]: Univ. Grenoble Alpes, Univ. Savoie Mont-Blanc, CNRS, LECA, 38000 Grenoble, France.

[9]: Univ. Limoges, INRA, PEREINE EA7500, USC1061 GAMAA, F-87000 Limoges, France.

[10]: CNRS, UMR 5815, Dynamiques du droit, Université Montpellier 1, France.

[11]: Utrecht University, Faculty of Veterinary Medicine, Utrecht.

Figure S1. NeighborNet graph based on F_{ST} genetic distance considering Moroccan and Algerian sheep breeds.

HAMAP=Hamra form pilot farms; HAMA=Hamra; BIGM=Beni-Guil; SDNA= Sidaoun; BRBA=Barbarine; BERA=Berber; OLDA= Ouled-Djellal from Algeria; OLDM=Ouled-Djellal from Morocco; DMNA=D'Man from Algeria; DMNM=D'Man from Morocco; SRDM=Sardi; TMHM=Timahdite; RMBA=Rembi; TZGA=Tazegzawth.

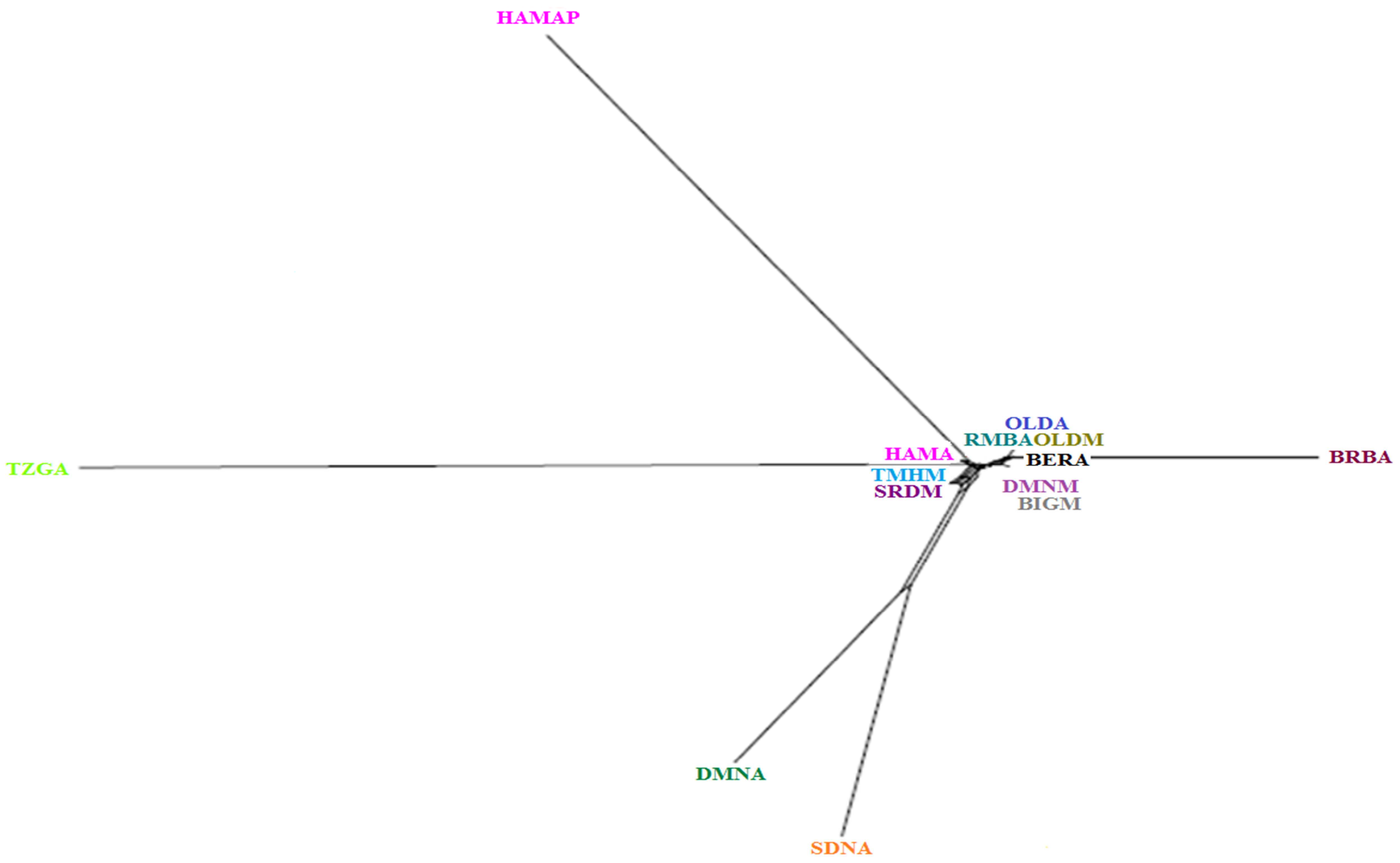

Figure S2. Individual tree based on ASD distance considering Moroccan and Algerian sheep breeds.

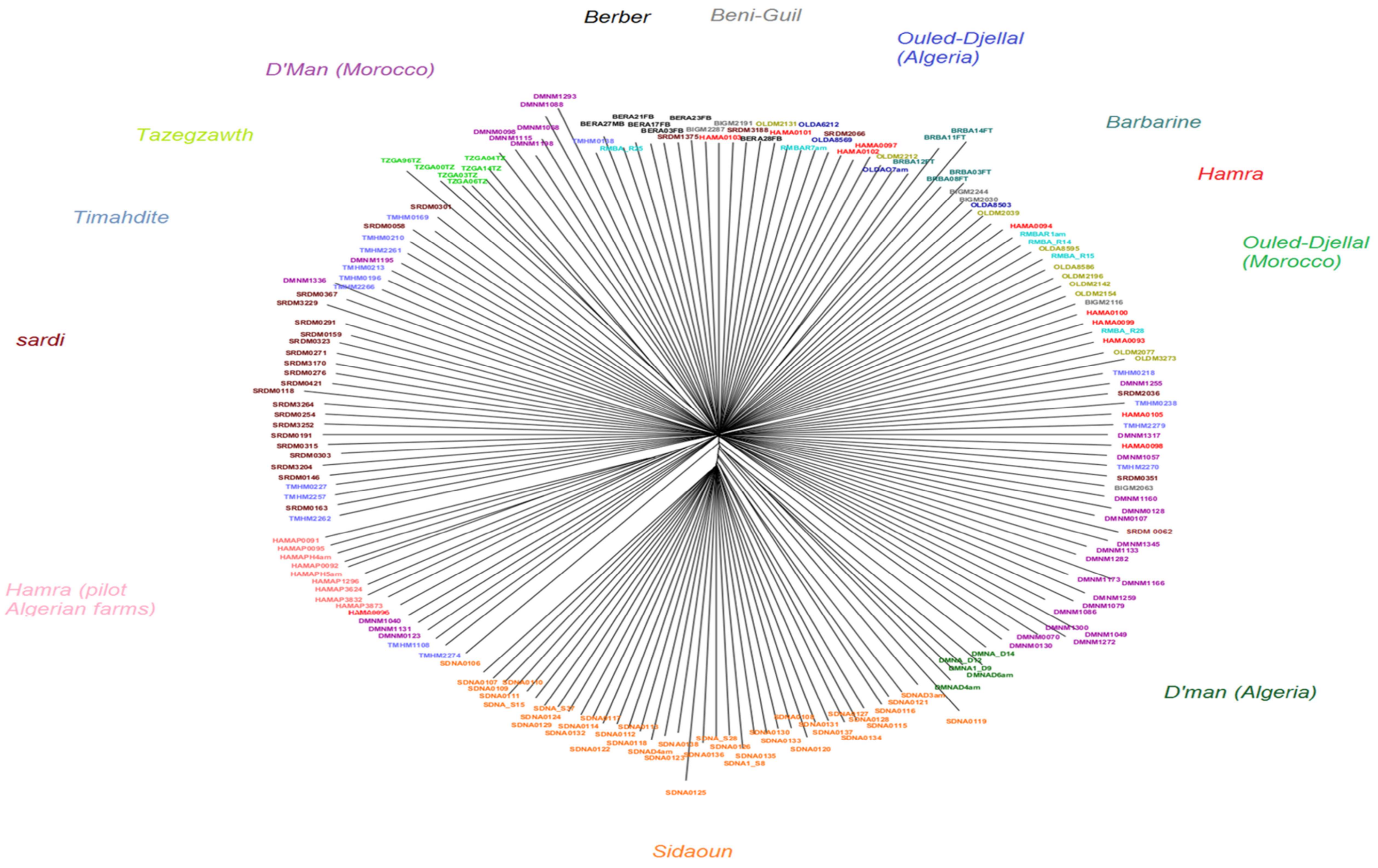

Figure S3. Distribution of the considered Maghrebin breeds around their cradle as described by Chellig[34] for Algeria by Boujenane[35] for Morocco.

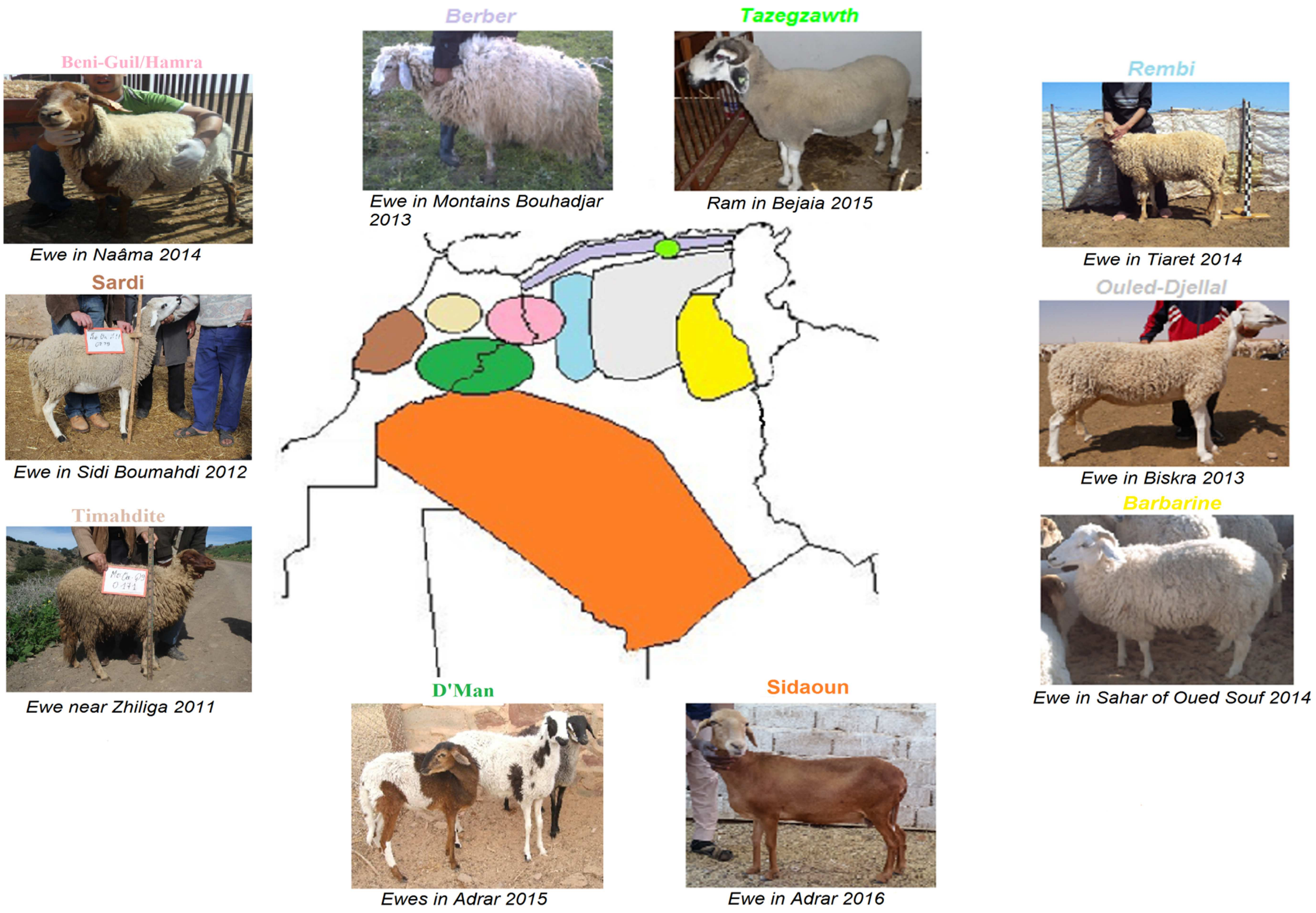

Table S1. Breed details for Algerian and Moroccan sheep breeds.

**Breed name**<br>**(code, country, nb.)**	**Phenotypic descriptors**	**Origin/Type**	**Distribution**	**Population size**	**Adaptative traits**
Ouled-Djellal<br>(Algeria=OLDA: 6, Morocco=OLDM: 8)	wool thin-tailed head and body uni-coloured (white)	Arab type<br>introduced into the country during the Zenete invasions (1,2); or by the Romans (3)	Eastern Algeria stretching across to Wadi Touil on the Tunisian border	11 340 000(for Algeria: 2003 FAO DAD-IS)	steppe and semi-steppe conditions (adapted to long walks)
Rembi<br>(Algeria=RMBA : 6)	wool thin-tailed long-legged<br>body uni-coloured (white)	Arab type	Chott Chergui in the west, Oued Touil in the east and the regions of Tiaret	2 000 000 (2003 FAO DAD-IS)	adapted to extreme cold, dryness, mountain's conditions and poor pastures
Timahdite<br>(Morocco=TMHM: 16)	wool thin-tailed body uni-coloured (white) and brown face	Arab origin supposed (4)<br>composite (Beni Guil and Tadla, breeds) (4)	Middle Atlas, mainly Timahdite and Ain Leuh	1 500 881 (1996 FAO DAD-IS)	well adapted to a wide range of pastoral and mixed farming environments
Barbarine<br>(Algeria=BRBA: 5)	Wool fat-tail	related to the Tunisian Barbarine introduced about 400 BC and later re-introduced (900 AD) by Arabs from the Near East of Asia (5)	spread throughout the east of the country, from the Oued Souf oasis to the border of Tunisia	48 600 (2003 FAO DAD-IS)	adaptive capacity to alternation of underfeeding and re-feeding periods
Sardi<br>(Morocco=SRDM: 27)	wool thin-tailed open spiral shaped horns	Arab origin supposed (4)<br>Crossing with the Tadla (4)	central Plateau and in the Tadla area	21 542 (1997 FAO DAD-IS)	well adapted to walking

Hamra/Beni-Guil<br>(Algeria: 11 from private farm=HAMA and 9 from the pilot farm=HAMAP)<br>Morocco=BIGM : 6)	wool thin-tailed medium legged	Berber (1) with Arab blood (6)	Steppes and high plaines of Algeria (Tlemcen, El-Bayedh, Saida, Mecheria and Ani Sefra) Plateaux of eastern Morocco	55 800 (for Algeria: 2004 FAO DAD-IS)	adapted to cold, wind and droughts
Tazegzawth<br>(Algeria=TZGA: 6)	wool thin-tailed peculiar black-spotted fleece; bluish tones muzzle, ears and around the eyes	unknown	Kabylie Mountains of Algeria	less than 2 200 (FAO 2014)	high adaptation to mountains' conditions
Berber<br>(Algeria=BERA: 6)	wool thin-tailed small conformation	Berber type (ancestral breed) (1)	Kabylie Mountains of Algeria	450 000 (2004 FAO DAD-IS)	rustic breed, suited to poor pasture
D'Man<br>(Algeria=DMNA: 5, Morocco=DMNM: 30)	mixed hair-wool sheep, thin-tailed short legged	Likely ancestry contribution from the forest hair sheep of West Africa (4)	raised almost permanently in the oases: Wadi Saorua Valley of south Algeria in the Sahel, valley of Ziz and the plain of Tafilalet in south Morocco	34 200 (for Algeria: 2004 FAO DAD-IS)	adapted to Sahelian conditions and high prolificacy
Sidaoun<br>(Algeria=SDNA: 39)	Hairy thin-tailed Short legged, very long tail	Originated from Mali exploited under nomadic conditions by the Tuareg people (7)	High Saharan, Tassili, Hoggar mountains and plains including Sahara ranges and the Oasis	23 400 (2004 FAO DAD-IS)	adapted to local Saharan climate (adapted to long walks)

(1) Sagne, J. *L'Algérie pastorale, ses origines, sa formation, son passé, son présent, son avenir*. Préface de Pierre Jore d'Arces. Editeur : Alger, Imprimerie Fontana (1950).

(2) Turries, V. *Les populations ovines algériennes*. Chaire de zootechnie et de pastoralisme, 16p. INA, Alger (1976).

(3) Trouette, M. *La sélection ovine dans le troupeau indigène*. Direction des Services de l'Élevage. Imprimerie P. Guiauchin, Alger, 1-10 (1933).

(4) Guessous, F., Rihani, N., Kabbali, A. & Johnson, W. L. Improving feeding systems for sheep in a Mediterranean rain-fed cereals/livestock area of Morocco. Journal of animal science (1989).

(5) Sanson, A. Les moutons. Histoire naturelle et zootechnie. Quatrième édition. Ouvrage orné de 56 gravures. Paris, Librairie agricole de la maison rustique (1885).

(6) Mason I.L. A Dictionary Of Livestock Breeds (1969).

(7) Lahlou-Kassi, A. *et al.* Performance of D'Man and Sardi sheep on accelerated lambing I. Fertility, litter size, postpartum anoestrus and puberty. *Small Rumin. Res.* (1989).

Table S2. Genomic inbreeding derived from ROH coverage (F_{ROH}) considering Algerian and Moroccan sheep breeds.

Breed	Number of animals	F_{ROH}<0.01 (%)	0.01<F_{ROH}<0.1 (%)	F_{ROH} >0.1 (%)
Sidaoun	39	26 (67%)	10 (26%)	3 (8%)
Berber	6	5 (83%)	0 (0%)	1 (17%)
Rembi	6	6 (100%)	0 (0%)	0 (0%)
Barbarine	5	2 (40%)	1 (20%)	2 (40%)
D’Man from Algeria	5	3 (60%)	2 (40%)	0 (0%)
Ouled-Djellal from Algeria	6	5 (83%)	1 (17%)	0 (0%)
Hamra	11	10 (91%)	1 (9%)	0 (0%)
Hamra from Algerian pilot farms	9	0 (0%)	9 (100%)	0 (0%)
Tazegzawth	6	0 (0%)	2 (33%)	4 (67%)
Beni-Guil	6	6 (100%)	0 (0%)	0 (0%)
Sardi	27	16 (59%)	8 (30%)	3 (11%)
Ouled-Djellal from Morocco	8	6 (75%)	1 (13%)	1 (13%)
D’Man from Morocco	30	14 (47%)	3 (10%)	13 (43%)
Timahdite	16	14 (88%)	2 (13%)	0 (0%)

Text S1. Analysis of segments of identity by descent (IBD) within 14 Italian local sheep breeds.

Dataset:

We considered 14 Italian local breeds genotyped by the BiOvIta Consortium (Ciani *et al.*, 2014) using the OvineSNP50K Genotyping BeadChip. ADMIXTURE was run for K=2 through K=20 and ten independent runs were performed for each value of K. The program CLUMPAK, was used to analyze the multiple independent runs at a single K and visualize the results. Fig. 1 displays results for K4, K8, K10 and K15.

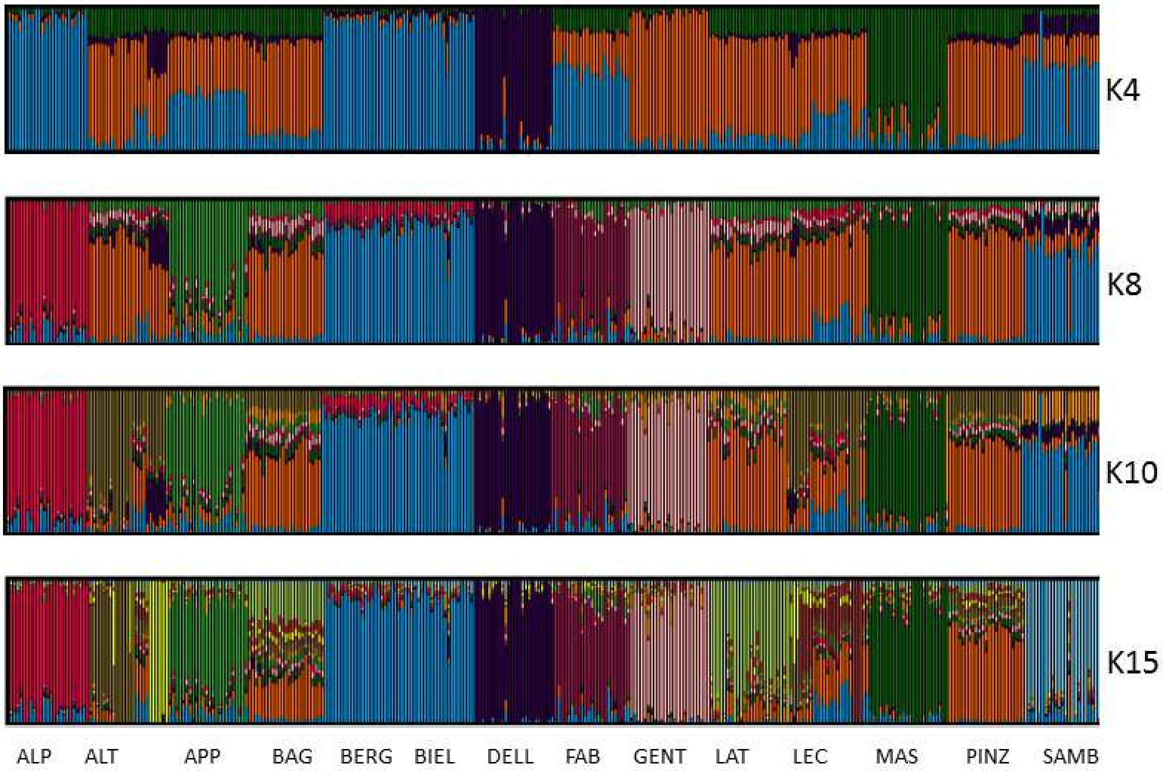

Figure 1. Bayesian clustering performed with ADMIXTURE software on Italian local sheep breeds. K = number of clusters.

For K15, proportion of membership for each predefined breed in each cluster was annotated (Table 1). These results allowed defining two groups:

(i) "Admixed" breeds, were defined as breeds sharing a cluster with one or several other breeds for more than 10% of membership: BERG, BIEL, LECC, LAT, BAG and PINZ

(ii) "Pure" breeds: ALP, ALT, APP, DELL, FAB, GENT, MASS and SAMB.

	K1	K2	K3	K4	K5	K6	K7	K8	K9	K10	K11	K12	K13	K14	K15
Alp	0.07	0.00	0.01	0.00	0.01	0.01	0.01	0.01	0.01	0.01	**0.84**	0.00	0.01	0.01	0.00
Alt	0.02	0.02	0.01	0.01	0.02	0.01	0.01	**0.30**	0.01	**0.47**	0.01	0.07	0.02	0.01	0.01
App	0.06	0.03	0.03	0.01	0.00	**0.72**	0.01	0.01	0.01	0.01	0.01	0.04	0.02	0.02	0.02
Bag	0.02	**0.28**	0.05	0.04	0.03	0.04	0.01	0.04	0.02	0.06	0.01	**0.29**	0.07	0.02	0.02
Berg	**0.87**	0.01	0.01	0.00	0.00	0.01	0.01	0.00	0.00	0.01	0.03	0.01	0.01	0.02	0.01
Biel	**0.85**	0.01	0.01	0.00	0.01	0.01	0.04	0.00	0.02	0.00	0.01	0.01	0.01	0.01	0.01
Dell	0.01	0.01	0.00	0.01	0.00	0.01	0.01	0.02	**0.90**	0.00	0.01	0.00	0.01	0.00	0.01
Fab	0.06	0.01	0.02	0.02	0.01	0.04	0.01	0.01	0.01	0.01	0.01	0.02	0.01	**0.75**	0.01
Gent	0.01	0.01	0.01	**0.83**	0.00	0.01	0.01	0.01	0.01	0.01	0.01	0.01	0.03	0.00	0.04
Lat	0.01	**0.70**	0.02	0.02	0.01	0.02	0.01	0.02	0.00	0.03	0.01	0.08	0.03	0.01	0.03
Lecc	**0.12**	0.03	0.02	0.01	**0.31**	0.03	0.02	0.08	0.01	0.09	0.02	0.20	0.04	0.01	0.01
Mas	0.03	0.03	**0.81**	0.01	0.01	0.02	0.01	0.01	0.01	0.01	0.01	0.01	0.01	0.01	0.01
Pinz	0.01	0.04	0.04	0.04	0.03	0.03	0.03	0.03	0.02	0.06	0.01	**0.60**	0.04	0.01	0.01
Samb	0.10	0.01	0.01	0.01	0.00	0.01	**0.76**	0.01	0.02	0.01	0.01	0.02	0.01	0.01	0.01

Table 1. Membership proportion for each predefined breed in 15 clusters. In bold proportion>0.1.

BEAGLE 4.1 was used for detection of IBD segments. The ibdtrim parameter was set to 40. Segments with a LOD score of <4 and a length shorter than 0.5 cM were excluded (Table 2).

Mean number of IBD segments per individual was significantly lower in “admixed” breeds (Wilcoxon rank sum test, p-value=0.001), with an average value of 83.64 in “pure” breeds against 12.72 in “admixed” breeds.

In particular, the consideration of two breeds highly admixed (Bergamasca (BERG) and Biellese (BIEL)), revealed that IBD segment length was significantly lower for these breeds, with a mean value of 7.38 Mb, compared to the other breeds, with a mean value of 12.52 Mb, (Wilcoxon rank sum test, p-value<0.001).

Breeds	Nb. of animals	Nb. of IBD segments	Nb. of IBD segment/Nb animals	% IBD segment intra-breed	% IBD segment inter-breed	Mean length (SD) Mb
“Pure” breeds						
Alpagota (ALP)	24	1398	58.25	100	0	10.32 (7.65)
Altamurana (ALT)	23	1657	72.04	70	30	12.78 (11.33)
Appeninica (APP)	24	1915	79.79	93.36	6.64	11.09 (9.24)
Delle Langhe (DELL)	23	3410	148.26	98	2	11.28 (9.43)
Gentile di Puglia (GENT)	24	2993	124.70	99	1	14.81 (13.59)
Sambucana (SAMB)	24	944	39.33	100	0	10.81 (9.0)
Massese (MAS)	23	1307	56.82	100	0	11.99 (9.47)
Fabrianese (FAB)	23	2069	89.95	100	0	13.28 (12.41)
“Admixed” breeds						
Bergamasca (BERG)	24	267	11.125	54.68	45.32	8.09 (5.53)
Biellese (BIEL)	22	155	7.04	55.59	44.41	6.67 (2.61)
Pinzirita (PINZ)	24	449	18.70	100	0	14.46 (12.33)
Laticauda (LAT)	21	337	16.04	97	3	10.59 (8.73)
Leccese (LEC)	24	316	13.16	100	0	13.67 (13.40)
Bagnolese (BAG)	22	226	10.27	83.19	16.81	15.11 (13.23)

Table 2. Analysis of IBD segments (number and length) considering Italian breeds. Nb., number; SD, Standard Deviation.

Reference:

Ciani, E. *et al.* Genome-wide analysis of Italian sheep diversity reveals a strong geographic pattern and cryptic relationships between breeds. *Animal Genetics* **45**, 256–266 (2014).

Text S2. Analysis of segments of identity by descent (IBD) within six South-West Asian sheep breeds.

Dataset:

We considered 6 local breeds from Turkish and Iran (Kijas *et al.*, 2012) using the OvineSNP50K Genotyping BeadChip. ADMIXTURE was run for K=2 through K=10 and ten independent runs were performed for each value of K. The program CLUMPAK, was used to analyze the multiple independent runs at a single K and visualize the results. Fig. 1 displays results for K2, K4, K6 and K8.

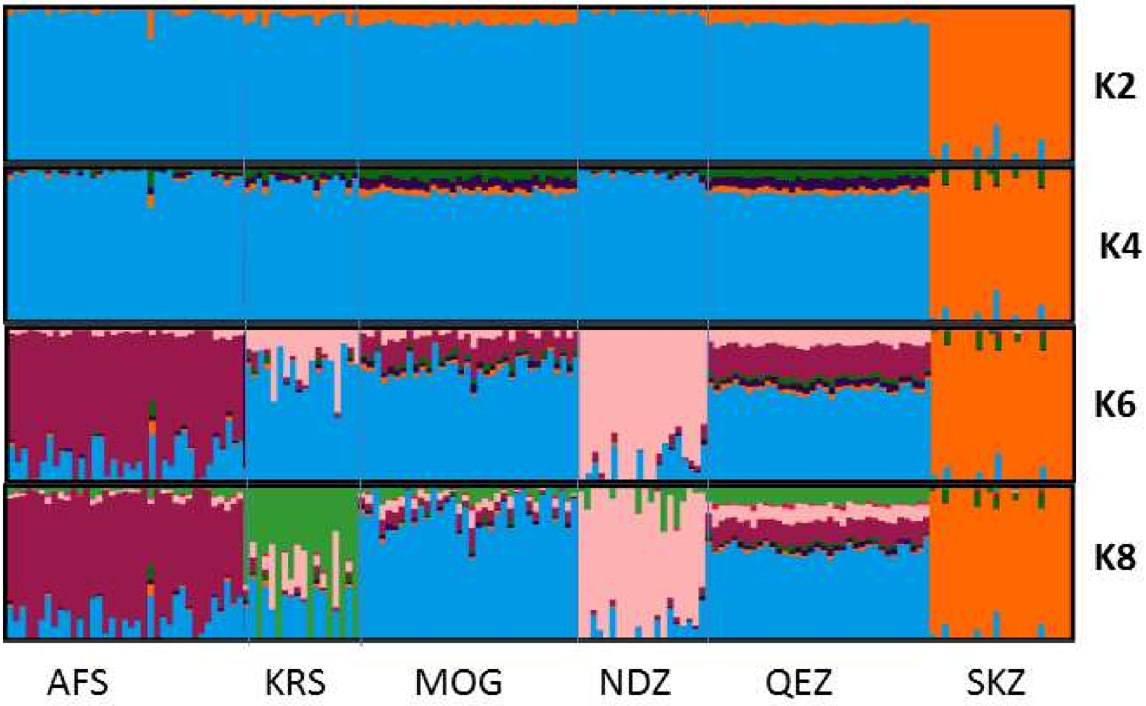

Figure 1. Bayesian clustering performed with ADMIXTURE software on South-West Asian local sheep breeds. K = number of clusters.

The dataset provided the opportunity to compare the case of MOG and QEZ, highly admixed, to the other breeds. Moreover SKZ was the only breed that did not share any cluster with the other breeds (for more than 10% of membership).

BEAGLE 4.1 was used for detection of IBD segments. The ibdtrim parameter was set to 40. Segments with a LOD score of <4 and a length shorter than 0.5 cM were excluded (Table 2).

Mean number of IBD segments per individual was significantly lower for MOG and QEZ (Wilcoxon rank sum test, p-value<0.001), with an average value of 6.58 for these highly admixed breeds, against an average value of 204.29 considering all of the others.

In particular, mean IBD segment length was significantly higher (Wilcoxon rank sum test, p-value<0.001) for SKZ (value of 19.99 Mb) compared to the other breeds (with an average value of 12.52 Mb).

Breeds	Nb. of animals	Nb. of IBD segments	Nb. of IBD segment/Nb animals	% IBD segment intra-breed	% IBD segment inter-breed	Mean length (SD) Mb
AFS	37	5759	155.64	100	0	11.93 (10.51)
KRS	18	2612	145.11	65.42	34.58	14.51 (14.35)
MOG	34	380	11.17	100	0	13.48 (14.23)
NDZ	20	2817	140.85	100	0	13.62 (12.87)
QEZ	35	70	2	100	0	13.86 (10.22)
SKZ	22	7121	323.68	100	0	19.99 (20.63)

Table 1. Analysis of IBD segments (number and length) considering South-West Asian sheep breeds. Nb., number; SD, Standard Deviation.

Reference:

Kijas, J.W. *et al.* Genome-Wide Analysis of the World's Sheep Breeds Reveals High Levels of Historic Mixture and Strong Recent Selection. *PLOS Biology* **10**, e1001258 (2012).
